# Same Day Discharge with Vaginal Packing Following Pelvic Floor Reconstruction: An Analysis of Safety and Healthcare Utilization

**DOI:** 10.1007/s00192-025-06186-y

**Published:** 2025-05-31

**Authors:** Tyler Trump, Omer Anis, Howard B. Goldman

**Affiliations:** Glickman Urological Institute, Lerner College of Medicine, Cleveland Clinic, Cleveland, OH USA

**Keywords:** Pelvic organ prolapse, Healthcare utilization, Healthcare economics, Vaginal packing

## Abstract

**Introduction and hypothesis:**

Vaginal packing is sometimes placed during pelvic floor reconstructive surgery to aid with hemostasis. Historically, these patients were admitted overnight. In the interest of moving patients safely and efficiently through the discharge process select patients are discharged home with vaginal packing. The objective of this study is to assess healthcare utilization and safety among patients discharged with packing.

**Methods:**

Retrospective review of patients undergoing pelvic organ prolapse (POP) surgery by a single surgeon between 2016 and 2023. Patients were identified before and after the 2020 COVID pandemic as this marked a transition point where same day discharge became heavily emphasized. The historic cohort (group 1) represents patients admitted overnight with vaginal packing compared to those discharged home same day to remove their own packing (group 2). Healthcare utilization and complications were recorded in the first 30 days postoperatively.

**Results:**

Thirty-eight patients were identified in group 1 and 39 in group 2. Age, BMI, and estimated blood loss was similar. There were 20 total unplanned encounters with 10 in each group (*p* = 0.95). Unplanned encounters in group 1 were seven phone calls/messages, one office visit, and two ED visits compared to three phone calls/messages, five office visits, and two ED visits in group 2. Overall complication rate was similar with six in group 1 and seven in group 2 (*p* = 0.80). There were zero cases of retained packing.

**Conclusion:**

Patients may safely be discharged home with vaginal packing in place with similar rate of complications and healthcare utilization when compared to hospital admission.

## Introduction

Pelvic organ prolapse (POP) is an increasingly prevalent condition with surgical volume for POP repair expected to increase by nearly 50% over the next four decades [1]. Vaginal packing is sometimes placed following pelvic floor reconstructive surgery especially in cases suspected to have persistent venous oozing following closure. Historically, these patients were admitted to the hospital overnight and packing was removed the following day [2]. More recently, there has been a shift to performing these surgeries on an outpatient basis when there are no concerns regarding hemostasis [3]. In the interest of moving patients safely and efficiently through the discharge protocol we often discharge select patients with vaginal packing in place.

Safety of vaginal packing is a topic of interest due to adverse effects associated with retained packing. Retained vaginal packing is associated with malodorous discharge, infection, decreased confidence in the healthcare team among patients, and increased legal claims [4, 5]. Obstetric units have successfully implemented protocols to mitigate the risk of retained packing following vaginal deliveries [4]. To our knowledge, there is no literature surrounding the safety and utility of discharging patients with packing in place following reconstructive surgery. The objective of this study was to evaluate the safety of discharging patients home with vaginal packing in place.

## Methods

We performed a retrospective analysis of patients undergoing POP surgery between January 2016 and December 2023 by a single surgeon. The study was approved by the institutional quality improvement chair. The patients were then further divided between two groups: those undergoing surgery prior to March 2020 (group 1) and those undergoing surgery after March 2020 (group 2). March 2020 designates the beginning of the COVID-19 pandemic in the United States at which time outpatient surgery was emphasized in an effort to maximally utilize inpatient hospital beds for patients affected by COVID-19. Prior to the COVID pandemic routine practice was to admit these patients postoperatively. Following the pandemic, patients were discharged home same day unless there were medical or surgical reasons to admit the patient.

We selected patients who underwent complex transvaginal native tissue prolapse repairs and had vaginal packing placed to remain until postoperative day (POD) 1. Patients were excluded if they had no packing placed or had packing placed that was removed on POD 0. Patients undergoing abdominal reconstructive procedures were excluded.

At the conclusion of the case, a single surgeon used his discretion whether to place packing or not based on his experience and expertise. At this time the duration of packing was also decided—to be removed in the postoperative area or to remain in place until POD 1. Those who were admitted had their packing removed the following morning prior to discharge. Patients discharged POD 0 with packing in place were given instructions for removal of packing. On the basis of the counseling and discussion, the patients decided whether they were comfortable removing their own packing and catheter or wished to have them removed in the clinic. They were encouraged to remove them at home. When packing was utilized, it was in the form of moistened rolled gauze placed tightly within the vaginal cavity. A foley catheter was left in place for all patients receiving packing for the duration of the packing.

Retrospective chart review was utilized to determine timing of packing removal, unplanned encounters within the first 30 days of the operation, and any postoperative complications. Unplanned encounters included any patient messages, telephone calls, office visits, and emergency department visits that deviated from their normal postoperative follow-up plan. Statistical analysis was performed using SPSS version 29.

## Results

Between January 2016 and December 2023 there were 407 transvaginal native tissue repairs performed (214 in group 1 and 193 in group 2). Within these cohorts, 38 patients were identified in group 1 and 39 in group 2 meeting inclusion criteria for overall rate of overnight vaginal packing of 17.8% and 20.2%, respectively (*p* = 0.53). The two groups were similar in age with a mean age of 71.7 years in group 1 and 73.8 years in group 2 (*p* = 0.31). The mean BMI was 27.6 in group 1 and 28.9 in group 2 (*p* = 0.29). Estimated blood loss was similar with a mean of 46.7 mL in group 1 and 39.2 mL in group 2 (*p* = 0.41). Preoperative comorbidities were assessed using patient’s preoperative American Society of Anesthesiologists (ASA) physical classification score. The groups were noted to be similar with regard to their comorbid conditions with a mean ASA score of 2.5 in group 1 and 2.6 in group 2 (*p* = 0.60). The most common surgical procedure performed in each group was a combined anterior and posterior repair with sacrospinous ligament fixation and levator myorrhaphy. The proportion of patients undergoing surgery involving a levator myorrhaphy was higher in group 2 (33/39, 84.6%) than in group 1 (22/38, 57) (*p* = 0.01) (Table 1). Within group 1 38/38 patients had their packing removed POD 1 prior to discharge. In group 2, 35/39 patients chose to remove their packing at home and 4/39 chose to have their packing and foley catheter removed in the office POD 1. All patients in group 2 had a documented phone call within 48 h postoperatively to inquire about packing and catheter removal.

**Table 1:** Illustrates patient demographics and surgical procedures performed

Age	71.1 ± 9.2	73.8 ± 7.1	*p* = 0.31
BMI	27.6 ± 5.3	28.9 ± 5.1	*p* = 0.29
EBL	46.7 ± 38.6	39.2 ± 40.7	*p* = 0.41
ASA class	2.5 ± 0.5	2.6 ± 0.6	*p* = 0.60
Surgery			
A and/or P	2	5	*p* = 0.25
SS ± A/P	27	29	*p* = 0.74
Colpocleisis	9	5	*p* = 0.22
Additional LM	22	33	***p* = 0.01**

*BMI* body mass index, *EBL* estimated blood loss, *ASA* American Society of Anesthesiologists, *A* anterior repair, *P* posterior repair, *SS* sacrospinous ligament fixation, *LM* levator myorrhaphy

There were 20 total unplanned encounters with 10 in each group (*p* = 0.95). The distribution of unplanned encounter types was similar with seven phone calls/messages, one office visit, and two ED visits in group 1 compared to three phone calls/messages, five office visits, and two ED visits in group 2 (*p* = 0.12). Overall complication rate was similar and there were six in group 1 and seven in group 2 (*p* = 0.80). Complications in group 1 included infection/UTI (3) and urinary retention (3). Complications in group 2 included hematoma (1), infection/UTI (1), pain (1), persistent vaginal bleeding (2), urinary retention (1), and catheter occlusion (1) (Table 2). There were zero cases of retained packing. With regard to unplanned encounters specifically related to the packing there was one encounter in group 1 (sensation of vaginal foreign body requesting exam) and three in group 2 (sensation of retained packing in 1 patient and persistent oozing requiring repacking in two patients) (*p* = 0.32).

**Table 2:** Illustrates surgical cases and patient characteristics with associated unplanned encounter and/or complication

	BMI	EBL	Case	Event	Unplanned encounter	Complication
Group 1	26.13	10	AP, LM	Inability to urinate, office visit POD 7 for catheter replacement with subsequent successful trial. Also requested exam for concern for foreign material in vagina-normal exam	Yes	Yes
	39.07	20	APSS	Telephone call POD 11 for persistent urinary frequency. Reassurance provided	Yes	No
	25.52	25	APSS	Telephone call POD 19 with complaint of fatigue and vaginal tenderness. Reassurance provided	Yes	No
	16.31	100	Colpocleisis,LM	Presented to ED POD 7 with urinary retention and concern for vaginal bleeding, catheter replaced with later successful trial	Yes	Yes
	23.3	10	Colpocleisis, LM	Treated at routine post-op visit for skin infection	No	Yes
	33.16	50	ASS	Telephone call POD 9 for bladder spasms. Reassurance provided	Yes	No
	28.15	40	APSS	Telephone call POD 16 for vaginal discharge. Treated for vaginal infection	Yes	Yes
	33.16	50	ASS	Telephone call POD 8 for bladder spasms. Reassurance provided	Yes	No
	28.15	40	APSS	Telephone call POD 17 for vaginal discharge. Treated for vaginal infection	Yes	Yes
	17.19	25	Colpocleisis, LM	Telephone call POD 8 for perineal discomfort. Reassurance provided	Yes	No
	30.83	50	APSS, LM	Presented to ED POD 16 with retention requiring catheter. Subsequent successful trial	Yes	Yes
Group 2	22.85	100	AP, LM	Fever POD 5 prompting ED visit. CT scan revealed self limited hematoma	Yes	Yes
	36.03	150	APSS, LM	Phone call POD 15 with UTI symptoms. Treated for UTI	Yes	Yes
	29.76	25	APSS, LM	POD 7 had sensation of foreign body concern for retained packing. Office visit confirmed no packing	Yes	No
	21.4	75	Colpocleisis, LM	Telephone call POD 13 for diarrhea, no intervention required	Yes	No
	26.38	10	APSS, LM	Telephone call for bothersome pain POD 12 requesting exam which was performed. Reassurance provided	Yes	Yes
	33.55	75	AP, LM	Packing removed at home with persistent oozing, presented to office and packing placed for additional 48 h with resolution	Yes	Yes
	21.55	25	APSS, LM	Phone call POD 5 for urinary leakage. Reassurance provided	Yes	No
	19.52	40	APSS, LM	Inability to urinate POD 1. Office for catheter replacement and subsequent successful trial	Yes	Yes
	24.11	25	APSS, LM	Packing removed at home with persistent oozing, presented to office and packing placed for additional 48 h with resolution	Yes	Yes
	29.95	30	APSS, LM	Presented to ED POD 1 due to catheter not draining. Clinician removed catheter and packing	Yes	Yes

*BMI* body mass index, *EBL* estimated blood loss, *A* anterior repair, *P* posterior repair, *SS* sacrospinous ligament fixation, *LM* levator myorrhaphy, *POD* postoperative day, *ED* emergency department, *UTI* urinary tract infection

## Discussion

In this paper, we performed analysis of 77 patients who received vaginal packing postoperatively until POD 1. Of these patients, 39 were discharged home with packing in place and 38 were admitted overnight to the hospital. Our results indicate that all patients had their packing removed in a timely fashion with few adverse events. Previously, vaginal packing was placed routinely to reduce bleeding and infectious complications. Standard practice was to admit the patients to the hospital overnight with removal of packing and foley catheter on postoperative day 1. Prior studies have suggested that routine packing is not necessary, but placement at the discretion of the surgeon on a case-by-case basis is appropriate [2, 6, 7]. Indeed, packing was placed in the minority of cases performed during this time period (18.9% of cases). Our results indicate that, when packing is deemed necessary, patients may be safely discharged home to remove their own packing, obviating the need for hospitalization or prolonged stay in the post-anesthesia care unit.

Discharging patients home same day with packing in place did not result in increased unplanned healthcare encounters when compared to the historic cohort of 38 patients who were admitted postoperatively. Of the unplanned encounters, half represented patient phone calls or messages that did not require any further evaluation or intervention. There were two patients who removed their own packing who presented to the office due to concern about persistent venous oozing following removal. These patients were both successfully managed with a brief period of repacking followed by resolution of bleeding. This highlights one potential issue with at home removal compared to clinician removal. In patients who have packing removed by clinician, in theory there is a time period following removal allowing for observation to determine if there is any further oozing whereas patients who remove it themselves have to present for evaluation if there is any concern. Nonetheless, given the infrequency of this occurrence and the overall similar rate of complications and unplanned encounters between the groups, we believe that this risk is outweighed by additional benefits of same day discharge. Hamilton et al. recently demonstrated a similar complication rate in patients undergoing packing and catheter removal 3 h postoperatively compared to those undergoing removal on POD 1 [8]. Their study did not evaluate patients undergoing same day surgery and all patients were admitted postoperatively. Their findings demonstrate an overall low rate (1%) of treatment owing to ongoing vaginal bleeding.

In our experience, packing is most commonly necessary following a case involving a levator myorrhaphy (58% of cases in group 1 and 85% in group 2). The levator myorrhaphy poses a unique challenge with regard to achieving a hemostatic closure in comparison to other components of transvaginal prolapse procedures. There is often slow venous bleeding that results from the dissection and deep interrupted sutures through the well vascularized levator muscles and achieving a “watertight” skin closure in this area is challenging. In our experience, the mild venous oozing that often persists intra-operatively responds well to packing. In our practice, a levator myorrhaphy is commonly performed in conjunction with a tight anterior/posterior repair with or without apical suspension in patients who are not sexually active but cannot undergo a traditional colpocleisis (due to inequality between degree of anterior and posterior prolapse). The proportion of patients who underwent LM in each group was higher in group 2. We believe this reflects more selectivity with patients who require packing after implementing same day discharge. In the era of routine hospital admission, we believe that we utilized packing more frequently since the patient would be evaluated in person the following morning. With same day discharge and the potential complications associated with packing, we have become more selective with utilization.

While our results support that packing may be safely left on discharge home, we cannot overstate the importance of a standardized protocol to ensure safe removal. Retained foreign bodies can be associated with both pain and infectious complications [4, 5]. Therefore, counseling and patient follow-up is paramount. Our routine practice in these cases is either to provide the patient with both clear written and verbal instructions for removal at time of discharge or to arrange to have them seen in our clinic within 48 h based on their preferences. Additionally, all surgical patients are contacted via telephone roughly 48 h postoperatively for a status update which provides an opportunity to ensure that they were able to remove their packing and foley catheter. Thus far, with this protocol in place, we have not had any instances of retained packing.

Our study aimed to assess the safety of leaving vaginal packing in place upon discharge. The study was not powered to assess the necessity of vaginal packing and that was outside the scope of the study. Additionally, we did not assess cost savings. Interestingly, a study of this nature has demonstrated cost savings and fewer encounters in patients who removed their foley catheter at home rather than presenting for an in-office voiding trial [9]. On the basis of our results and similar rate of unplanned encounters, we would expect similar findings in patients discharged home with packing, but future studies would be required to validate this.

A limitation of this study is the retrospective design and reliance on the electronic medical record to provide information regarding postoperative course. For example, we could only capture events that happened within our healthcare system and patients who presented elsewhere for care in the specified study period may have been missed. Patient satisfaction was not studied and would provide valuable insight regarding patient preferences with both hospital admission versus same day discharge and also at home packing removal versus in office packing removal. We would expect patients to prefer at home packing removal since they are given both options prior to discharge but having data regarding their experience and preferences following the packing removal would be valuable for future patient counseling. Finally, we present our experience in a small sample size from a single surgeon. Further studies with larger sample sizes and in a variety of practice settings would be valuable.

## Conclusion

Same day discharge with vaginal packing in place is safe and patients can reliably remove their own packing at home. The need for vaginal packing should not discourage surgeons from following their same day surgery protocol in patients otherwise suited for outpatient surgery.

## Data Availability

Data is available upon reasonable request to the corresponding author.

## Declarations

### Institutional Review

The study was approved after review from the institute quality improvement chair as a quality improvement project.
